# Integrative analysis of RNA-sequencing and microarray for the identification of adverse effects of UVB exposure on human skin

**DOI:** 10.3389/fpubh.2024.1328089

**Published:** 2024-02-20

**Authors:** Yujin Jang, Hye-Won Na, Dong Yeop Shin, Jun Lee, Jun Pyo Han, Hyun Soo Kim, Su Ji Kim, Eun-Jeong Choi, Charles Lee, Yong Deog Hong, Hyoung-June Kim, Young Rok Seo

**Affiliations:** ^1^Department of Life Science, Institute of Environmental Medicine for Green Chemistry, Dongguk University Biomedi Campus, Gyeonggi-do, Republic of Korea; ^2^Research and Innovation Center, Amorepacific, Gyeonggi-do, Republic of Korea; ^3^National Institute of Environmental Research, Incheon, Republic of Korea; ^4^The Jackson Laboratory for Genomic Medicine, Farmington, CT, United States

**Keywords:** ultra violet-B, cutaneous melanoma, meta-analysis, batch effect, LIF, network analysis, toxicogenomics

## Abstract

**Background:**

Ultraviolet B (UVB) from sunlight represents a major environmental factor that causes toxic effects resulting in structural and functional cutaneous abnormalities in most living organisms. Although numerous studies have indicated the biological mechanisms linking UVB exposure and cutaneous manifestations, they have typically originated from a single study performed under limited conditions.

**Methods:**

We accessed all publicly accessible expression data of various skin cell types exposed to UVB, including skin biopsies, keratinocytes, and fibroblasts. We performed biological network analysis to identify the molecular mechanisms and identify genetic biomarkers.

**Results:**

We interpreted the inflammatory response and carcinogenesis as major UVB-induced signaling alternations and identified three candidate biomarkers (*IL1B*, *CCL2*, and *LIF*). Moreover, we confirmed that these three biomarkers contribute to the survival probability of patients with cutaneous melanoma, the most aggressive and lethal form of skin cancer.

**Conclusion:**

Our findings will aid the understanding of UVB-induced cutaneous toxicity and the accompanying molecular mechanisms. In addition, the three candidate biomarkers that change molecular signals due to UVB exposure of skin might be related to the survival rate of patients with cutaneous melanoma.

## 1 Introduction

Ultraviolet (UV) radiation from sunlight that reaches the Earth’s surface is one of the major environmental factors that cause toxic effects resulting in structural and functional cutaneous abnormalities in most living organisms (1). There are three types of UV radiation: UVC (100–280 nm), UVB (280–320 nm), and UVA (320–400 nm) (2). UVC is less relevant to the harmful effects of UV radiation on the skin because it is completely absorbed by stratospheric oxygen (2). The majority of UVB is absorbed by the ozone layer but still reaches ground level and is absorbed in sufficient amounts to have a deleterious impact on the skin (2, 3). UVA and UVB are reported to induce DNA damage, photoaging, and skin cancer (4, 5). However, UVA radiation is much less photochemically active and not strongly absorbed by proteins and nucleic acids, so it is generally less harmful than UVB radiation (2, 3). In humans, UVB exposure has the most damaging effects and acts as a tumor initiator by producing irreversible mutagenic damage to the epidermis through photoproducts, photo-hydrates, and oxidative damage (6, 7).

Skin cutaneous melanoma (SKCM) originates in melanocytes and is the most aggressive type of skin cancer with the highest mortality due to its metastatic potential. SKCM is currently the fifth and seventh most common cancer diagnosed in men and women, respectively in the United States. Its prevalence has increased faster than any other cancer within the previous 50 years (8). Given the high mortality risk and increasing prevalence of SKCM, it is important to screen biomarkers for the early diagnosis of SKCM. Several clinical parameters, including sex, age, family history of skin cancer, and skin phototypes, have been reported as risk factors for SKCM, but UV exposure is regarded as the main risk factor. It is estimated that 60–70% of SKCM is caused by UV radiation and several studies revealed that UVB exposure induced melanoma formation (9–11).

With the rapid development of biological high-throughput techniques, various gene expression data are continuously accumulated (12). Most studies associated with these data have limited statistical power due to relatively small sample sizes (12), but combining the information (a meta-analysis) increases the sensitivity and provides validated conclusions based on gene expression integration (13). The toxicogenomic research from a single study is limited to the specific predetermined experiment conditions (e.g., specific treatment concentrations, target organs or cells, and treatment period). In this regard, retaining the robustness of biomarkers or molecular mechanisms is a major challenge.

This study aimed to provide insights into the adverse effects of UVB on human skin and the biological association between UVB exposure and cutaneous manifestations through a meta-analysis of publicly available transcriptomic data. In this study, we attempted to combine multiple datasets from heterogeneous studies of UVB exposure on human skin, explore the molecular mechanisms, and identify potential biomarkers via an integrative interpretation of the gene expression profiles. Furthermore, we determined the association between the selected candidate biomarkers of UVB exposure and the survival probability of patients with SKCM.

## 2 Materials and methods

### 2.1 Data collection

The microarray and RNA-sequencing (RNA-Seq) datasets were downloaded from the Gene Expression Omnibus^1^ and Sequence Read Archive^2^ databases to obtain gene expression profiles using the keywords “UVB” and “UVB radiation.” Basic inclusion criteria were (1) gene expression profiles of human-derived skin samples (2). Experiments that exposed samples to UVB without other substances or drugs (3). Acute exposure experiments, which refer to contact with a substance that occurs once or for only a short time (14). Finally, (4) studies that provided processed RNA-Seq data where read count data must be accessible to prevent noise from the analysis results. The final selected datasets consisted of two microarray experiments and six RNA-Seq experiments, encompassing 39 control samples and 41 UVB-exposed samples. These datasets and their detailed information are summarized in Supplementary Table S1.

### 2.2 Data preprocessing of microarray and RNA-Seq datasets

The raw expression data of the Agilent microarray (GSE45493) and Affymetrix (GSE41078) microarray were normalized using the “normalizedBetweenArrays” function in the limma R package (15).

In the RNA-Seq data, raw data (FASTQ files) were downloaded from the SRA database, and low-quality reads were trimmed using Trimmomatic software (v0.36) (16). The trimmed reads were aligned to the human reference genome (GRCh38) using HISAT2 (v2.2.1) (17). SAMtools (v1.10) was used to convert the SAM file to a BAM file and sort the sequences (18). The read count data were generated by StringTie (v2.2.0) or downloaded from the GEO database (19). Low-count genes were filtered out, and normalization was performed using edgeR packages (v3.38.1) in R software (20). The expression level of RNA-Seq results was analyzed using counts per million (CPM) values.

### 2.3 Meta-analysis between the microarray and RNA-Seq

To integrate microarray and RNA-Seq data, the CPM values of RNA-Seq data were transformed (21, 22) using Eq. (1) to eliminate the singularity at CPM = 0.


(1) $$x\;=\;\log2\;\left(CPM\;+\;1\right)$$

After transformation, each dataset was annotated with an Ensembl ID and expression data were integrated based on common Ensembl gene IDs. Batch effects (non-biological differences) were adjusted by applying the “sva” function (v3.44.0) in R software (23).

### 2.4 Clustering analysis and differentially expressed genes screening

After batch-effect correction, the principal component analysis (PCA) was conducted to examine the distributions derived from different groups. Differentially expressed genes (DEGS) between the control and UVB-exposed groups were identified using the limma R package (v3.52.2). The threshold criteria were |fold change| ≥1.5, and false discovery rate adjusted *p* value < 0.05. The expression patterns of the DEGs were visualized using a hierarchical clustering heatmap.

### 2.5 Network analysis

Pathway Studio (v12.5.0.2), a commercial text mining-based analytical tool, was used to explore and visualize the various types of functional interactions between biological entities, including genes, cell processes, and diseases (24). Pathway Studio provides insight into the biological relationships between the entered genes, as well as an investigation of protein-functional class, protein-disease, or protein-cell process. In addition, to uncover the key entities that contribute significantly to the network, betweenness centrality and degree values were calculated using NetworkAnalyzer in Cytoscape (v3.8.2) (25). To construct a network based on more genetically significant genes, threshold criteria were narrowed (|fold change| ≥2). Finally, the top 45 genes were selected with a degree ≥3 based on the gene–gene interactions derived from the Pathway Studio database information. In addition, the signaling network was expanded to explore major cell processes, cutaneous diseases, and functional classes associated with selected genes. Twenty-nine cell processes, 21 skin diseases, and 5 functional classes were selected based on the criteria that the number of references should be at least 3 for all relations, and the degree value should be at least 3 for skin disease, 5 for cell process, and 10 for functional class.

### 2.6 Gene ontology pathway functional analysis of DEGs

To explore the potential function of the identified significant DEGs, gene ontology term analysis was performed using the clusterProfiler R package (v4.4.4) (26). The gene ontology or GO terms, refer to the following domains: biological process, cellular component, and molecular function. Enrichment analysis was performed with the threshold of adjusted *p* value < 0.05.

### 2.7 Survival analysis

A survival analysis was performed using Survival Genie (https://bbisr.shinyapps.winship.emory.edu/SurvivalGenie/), a web platform for survival analysis related to adult and pediatric cancers (27). Genes were uploaded to the Survival Genie web platform, and SKCM dataset in The Cancer Genome Atlas (TCGA) dataset was selected to perform the survival analysis. Metastatic tumor type and median-based partitioning method were selected. Kaplan–Meier survival curves were generated by the Survival Genie platform, and the survival rates of the stratified high (red) and low (blue) groups of patients were shown according to log-rank tests.

### 2.8 Cell culture and UVB exposure

Normal Human Epidermal Keratinocytes (NHEKs) and Normal Human Dermal Fibroblasts (NHDFs) were purchased from Lonza (Basel, Switzerland). NHEKs were cultured in keratinocyte growth medium (KBM-Gold) with SingleQuots™ supplement (Lonza), including bovine pituitary extract, epinephrine, gentamicin/amphotericin B, human epidermal growth factor, hydrocortisone, insulin, and transferrin at 37°C in a 5% CO_2_ incubator. NHDFs were cultured in Dulbecco’s modified Eagle medium (DMEM) containing 4.5 g L^−1^ glucose, L-glutamine, 10% fetal bovine serum, and 1% penicillin–streptomycin mixture (Gibco, United States) at 37°C in a 5% CO_2_ incubator. NHEKs and NHDFs were irradiated with UVB (312 nm) at various intensities (11–20 mJ cm^−2^) in phosphate-buffered saline using a Bio-Sun UV irradiation system (Vilber Lourmat). After UVB exposure, the NHEKs were incubated in KBM-Gold and the NHDFs were incubated in DMEM, both for 24 h.

Cell viability was measured to select an appropriate UVB intensity. NHEKs were irradiated at 13 mJ cm^−2^ based on the results of the cell viability test in our previous study (28). For NHDFs, the 3-(4,5-dimethylthiazol-2-yl)-2,5 diphenyltetrazolium bromide (MTT) assay was performed to confirm cell viability. NHDFs were seeded in 96-well cell culture plates and stabilized overnight at 37°C. NHDFs were shaded with aluminum foil and irradiated with 0, 11, 13, 15, and 19 mJ cm^−2^ UVB, respectively. After 24 h at the end of the exposure, the MTT assay was performed according to the manufacturer’s protocols.

### 2.9 RNA isolation and quantitative reverse transcription polymerase chain reaction

RNA from NHEKs and NHDFs was extracted and the quality control of RNA samples was performed as described previously (28). cDNA was synthesized and quantitative reverse transcription polymerase chain reaction (qRT-PCR) was performed as described previously (28). The primer sequences of four genes, *GAPDH*, *IL1B*, *CCL2*, and *LIF*, and the thermal cycling conditions are shown in Supplementary Table S2.

### 2.10 Statistics

Statistical analyses were performed using R software (v4.2.2). The differences between the control and exposure groups were analyzed using Student’s *t*-test with *p*-value <0.05. GO analyses were performed using R software, and adjusted *p*-value <0.05 were considered statistically significant. Statistical analysis of survival analysis was performed in Survival Genie software, and a log-rank *p*-value <0.05 was considered significant.

## 3 Results

### 3.1 Identification of gene lists associated with UVB exposure of skin

**Figure 1:**
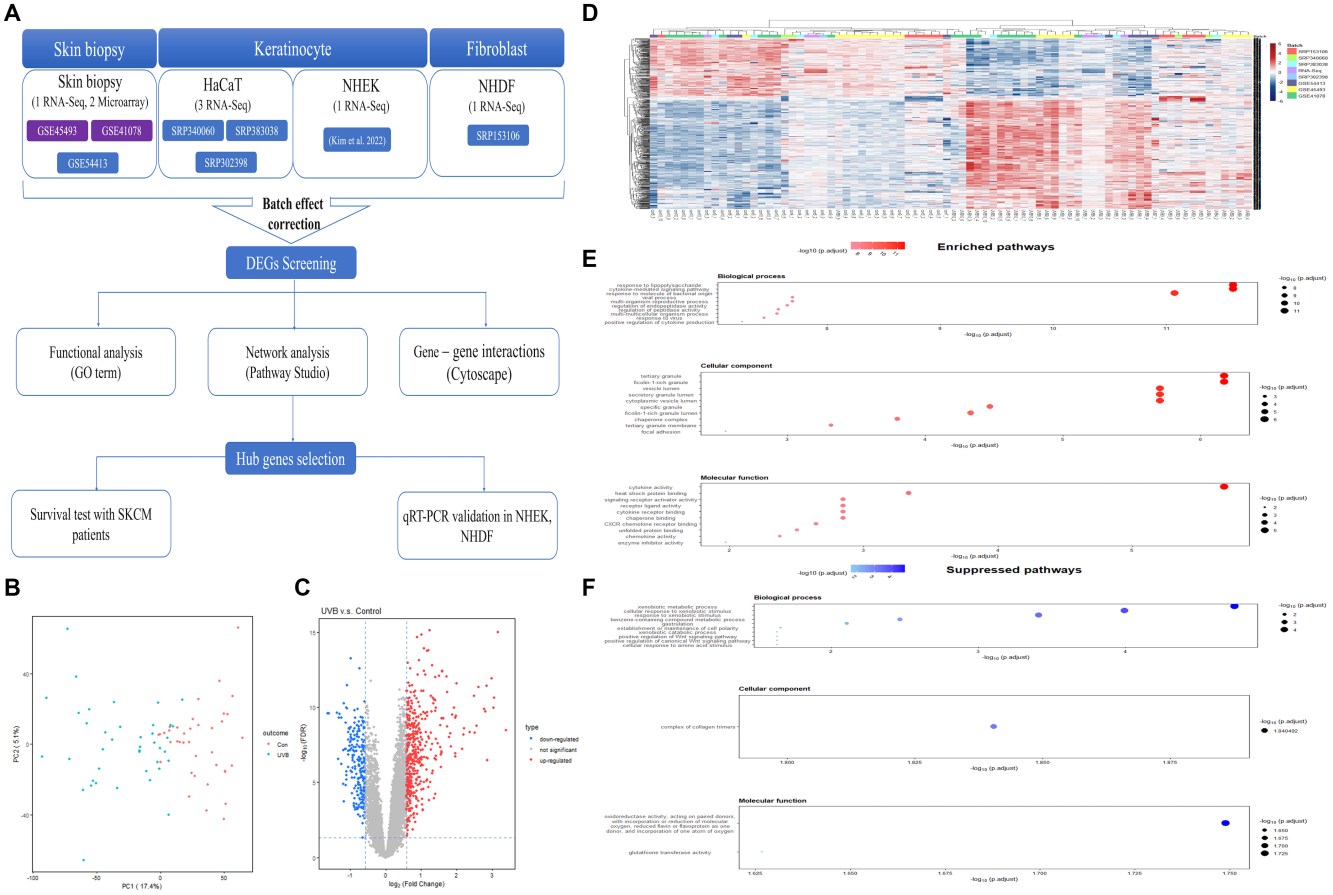
Workflow of a meta-analysis and expression profiles of DEGs in response to UVB exposure on the skin. **(A)** Workflow of a meta-analysis between microarray and RNA-Seq. **(B)** Batch effects-adjusted PCA result. **(C)** Volcano plot result of DEGs. **(D)** Hierarchical clustering heatmap of the DEGs. **(E)** Top 10 significantly upregulated GO terms of DEGs. **(F)** Top 10 significantly downregulated GO terms of DEGs. DEG, differentially expressed gene; UVB, ultraviolet B; GO, Gene Ontology.

To identify consistently regulated genes upon UVB exposure of skin, eight gene expression profile datasets were collected from the GEO and SRA databases considering the inclusion criteria. Specifically, six different RNA-Seq datasets were used, including one previously published dataset (28) and two microarrays. Furthermore, various skin cell types, such as skin biopsies, fibroblasts, and keratinocytes, were used to prevent biases toward single cell types. To overcome differences between the microarray and RNA-Seq platforms, the RNA-Seq data were slightly transformed (as detailed in the “Materials and methods”). As the merged expression dataset originated from various cell types and platforms, it was important to focus solely on the effect of UVB exposure. Therefore, the batch effects were adjusted for each cell type to focus on UVB exposure, as shown in Figure 1A.

### 3.2 Clustering patterns of meta-signatures profiles

The results of PCA indicated that UVB-exposed samples were mostly distinct from control samples with some exceptions, and most of the variances due to tissue origin or the platform of each dataset were not identified (Figure 1B). After the integration of eight studies, 681 robust genes that were differentially expressed were extracted (upregulated: 450, downregulated: 231; Figure 1C). There was an almost complete consistent distinction in the gene expression profiles between the control and UVB exposure groups (Figure 1D).

### 3.3 Go enrichment analysis of the significant DEGs

GO enrichment analysis was performed to investigate the potential functions of the obtained DEGs. Figure 1E and Supplementary Table S3 show which biological functions were mainly predicted by upregulated genes. In biological processes, the activated GO terms were confirmed to be related to the immune response, such as response to lipopolysaccharide, cytokine-mediated signaling pathway, and response to a molecule of bacterial origin. In cellular components, the main GO terms were related to intracellular organelles, including tertiary granule, ficolin-1-rich granule, vesicle lumen, secretory granule lumen, and cytoplasmic vesicle lumen. In molecular functions, cytokine responses, including cytokine activity and cytokine receptor binding, were the predicted enriched GO terms, and reactions to temperatures and external stimuli, including heat shock protein binding, were identified.

At the same time, we identified biological functions that were mainly predicted by downregulated genes (Figure 1F; Supplementary Table S4). In biological processes, the suppressed GO terms were confirmed to be related to xenobiotic response, including xenobiotic metabolic process, cellular response to xenobiotic stimulus, and response to xenobiotic stimulus. In cellular component, there was only one related GO term; the downregulated genes were mainly involved in the complex of collagen trimers. In molecular functions, the downregulated genes were mostly associated with two categories: (1) oxidoreductase activity, acting on paired donors, with the incorporation or reduction of molecular oxygen, and (2) glutathione transferase activity.

### 3.4 Dermatologic network associated with UVB exposure

**Figure 2:**
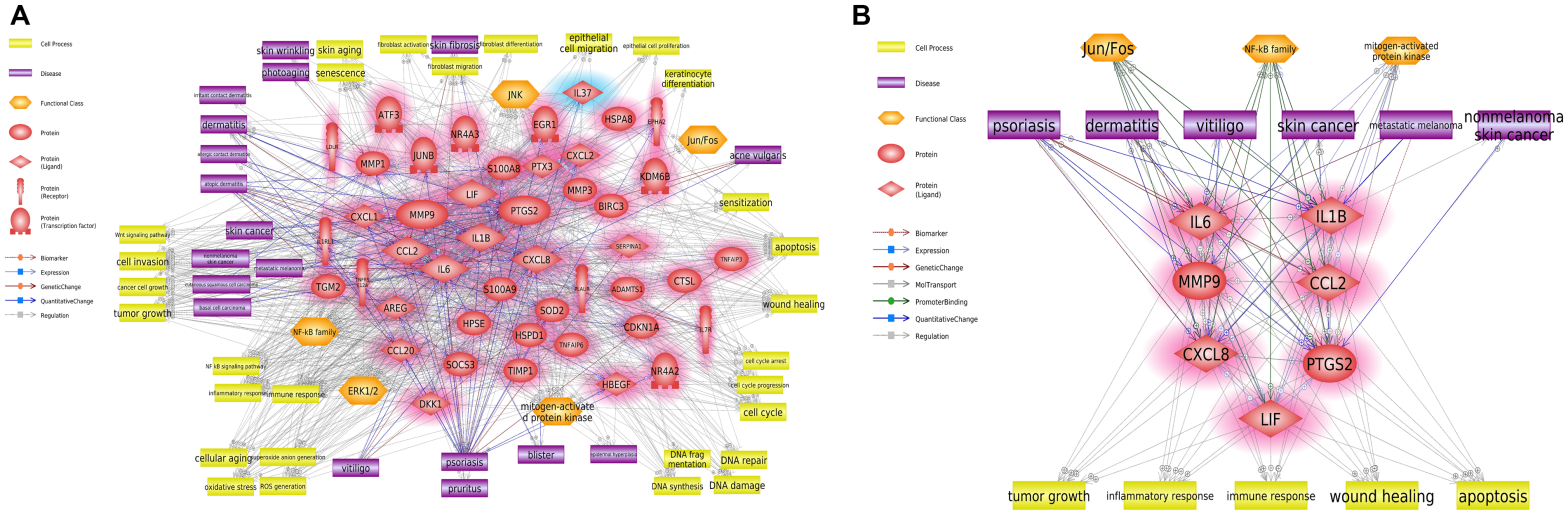
Biological networks for cutaneous effects of UVB exposure. **(A)** Biological network associated with UVB exposure of human skin. **(B)** Summarized hub network. Red and blue highlights indicate up- and downregulated DEGs, respectively. UVB, ultraviolet B; DEG, differentially expressed gene.

To explore the dermatologic signaling network and hub genes associated with UVB exposure, we used Pathway Studio software to perform network analysis of DEGs in response to UVB exposure. Based on the text-mining algorithm of the software, we expanded the signaling network to predict the biological functions that may be altered in UVB-exposed skin (Figure 2A). The genes significantly associated with inflammatory signaling were identified from functional classes, including NF-κB family, ERK1/2, mitogen-activated protein kinase, JNK, and Jun/Fos. In terms of cell processes, the network among the genes was closely related to altered carcinogenic processes, including tumor growth, cell invasion, and cancer cell growth. Inflammatory cell processes were also predicted, including inflammatory response and immune response. Association with cancer-related diseases was shown, including skin cancer, basal cell carcinoma, metastatic melanoma, and nonmelanoma skin cancer.

### 3.5 Hub network associated with UVB exposure of skin

We analyzed the biological hub network from Figure 2A to construct potential hub networks that might elucidate the biological relationships between cutaneous effects and UVB exposure (Figure 2B). Seven hub genes (*IL1B*, *IL6*, *MMP9*, *CCL2*, *CXCL8*, *PTGS2*, and *LIF*) and major entities were screened considering the betweenness centrality and degree value (Supplementary Table S5; Supplementary Figure S1). NF-κB, Jun/Fos, and mitogen-activated protein kinase were predicted to be the major functional classes. Tumor growth, inflammatory response, immune response, wound healing, and apoptosis were predicted to be the major cellular processes. In major diseases, psoriasis, dermatitis, vitiligo, skin cancer, metastatic melanoma, and nonmelanoma skin cancer were selected.

### 3.6 Verification of hub genes using qRT-PCR and identification of the survival probability of patients with cutaneous melanoma

**Figure 3:**
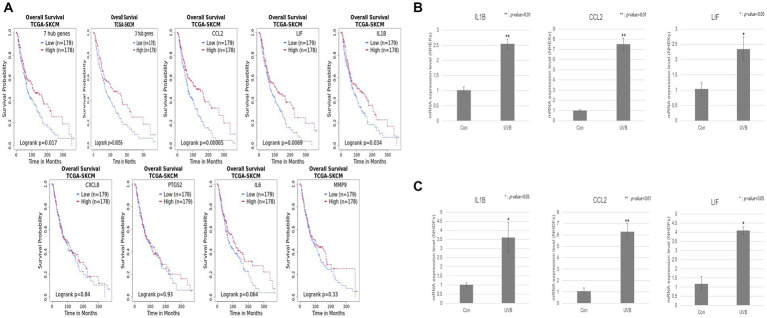
Validation of the hub genes by a survival analysis of patients with SKCM and qRT-PCR. **(A)** Kaplan–Meier curve plot of seven hub genes, three hub genes, and individual genes *CCL2*, *LIF*, *IL1B*, *CXCL8*, *PTGS2*, *IL6*, and *MMP9*. The red and blue lines represent the high- and low-expression groups, respectively. The vertical and horizontal axes represent the probability of survival and the survival time (months), respectively. **(B,C)** The relative mRNA expression levels of the three hub genes (*IL1B*, *CCL2*, and *LIF*) were detected by qRT-PCR in UVB-irradiated or normal keratinocytes and fibroblasts, respectively. Error bars represent ± SEM. * and ** indicate *p*-values <0.05 and < 0.01, respectively.

We performed a survival analysis to examine the correlation between the relative expressions of the seven hub genes and patients with metastatic SKCM. All patients with metastatic SKCM were divided into low- and high-expression groups according to the gene expression levels (Table 1). For the set of 7 hub genes, 179 low- and 178 high-expression groups were separated among 357 patients with SKCM. As shown in Figure 3A, the set of seven hub genes was considered to be significantly associated with the prognosis (*p* value < 0.05) of patients with SKCM. We found that *IL1B*, *CCL2*, and *LIF* among the seven hub genes were individually associated with the overall survival of patients with SKCM (*p* value < 0.05; Table 1; Figure 3A).

**Table 1:** Survival analysis of seven hub genes.

Gene symbol	*P*-value HR	Hazard ratio (95% CI)	Number of low cases	Number of high cases
*IL1B*	0.0349	0.74 (0.55–0.98)	179	178
*IL6*	0.0652	0.77 (0.58–1.0)	179	178
*MMP9*	0.33	0.87 (0.65–1.2)	179	178
*CCL2*	0.000733	0.61 (0.46–0.81)	179	178
*CXCL8*	0.835	0.97 (0.73–1.3)	179	178
*PTGS2*	0.93	0.99 (0.74–1.3)	178	179
*LIF*	0.00723	0.67 (0.51–0.9)	179	178
Set of seven hub genes	0.0173	0.71 (0.53–0.94)	179	178
Set of three hub genes	0.00589	0.67 (0.50–0.89)	178	179

To confirm the expression level of the three hub genes (*IL1B*, *CCL2*, and *LIF*) in keratinocytes and fibroblasts upon UVB exposure, we used NHDFs and NHEKs. The UVB exposure resulted in dose–response curves for both cell lines. UVB intensity showed >80% cell viability at an irradiation dose of 13 mJ cm^−2^ in NHEKs and 11 mJ cm^−2^ in NHDFs (Supplementary Figure S2). qRT-PCR was performed to confirm the RNA expression levels of hub genes under UVB exposure conditions. Among them, all three hub genes showed significant upregulation in UVB-irradiated NHDFs and NHEKs (Figures 3B,C).

## 4 Discussion and conclusion

UVB radiation is one of the most important environmental factors affecting the skin. The stratum corneum absorbs 70% of UVB radiation, 20% of which reaches the epidermal layer, which contains keratinocytes, melanocytes, and epidermal stem cells, and only 10% penetrates the uppermost part of the dermis layer where fibroblasts are located (29, 30). UVB irradiation can cause changes in cell function or DNA damage, leading to erythema, burns, and, eventually, skin cancer (31, 32). However, detailed knowledge of the molecular mechanisms of cutaneous disease caused by UVB radiation through the interpretation of genetic changes is still lacking. To address this gap, this study integrated and interpreted almost all accessible data on skin cell types exposed to UVB and suggested the toxic mechanisms and potential biomarkers.

To explore the UVB-induced signaling pathway, we acquired significant genetic profiles from all accessible public data (Figure 1A; Supplementary Table S1). Various skin cells were considered, not only skin biopsy data showing the overall skin effect but also keratinocytes, the most abundant cells in the epidermis and a major focus of research on UVB, and fibroblasts, which although they receive only a small fraction of the solar UVB, can mediate UVB-mediated damage and aging (33, 34). After adjusting for the batch effects of heterogeneous datasets, upregulated robust genes were mainly enriched in immune responses, including the cytokine-mediated signaling pathway, response to a molecule of bacterial origin, and positive regulation of cytokine production, from the functional enrichment analysis using GO analysis (Figure 1E). The inflammatory response is a series of complex and coordinated events triggered by the host to defend against extrinsic stimuli (35). UVB radiation is known to cause the release of inflammatory mediators from various skin cells and activate the immune cells in the skin (36). Our data also revealed that UVB exposure resulted in the downregulation of robust genes enriched in GO terms associated with xenobiotic processes, including xenobiotic metabolic process, response to xenobiotic stimulus, and xenobiotic catabolic process, as shown in Figure 1F. This suggested that the protective reaction to external substances tends to decrease after damage by UVB exposure (37).

In the network analysis results, we identified seven hub genes (*IL1B*, *IL6*, *MMP9*, *CCL2*, *CXCL8*, *PTGS2*, and *LIF*) in response to UVB-induced cutaneous effects (Figure 2). *IL1B* was the most strongly associated with the signaling network (Supplementary Table S5). This cytokine is an important mediator of the inflammatory response and is involved in various cellular activities, including cell proliferation, differentiation, and apoptosis (38). Elevated *IL1B* expression is associated with skin diseases, including inflammation and cancer, by inducing inflammasome activation (39, 40). *IL6* has a role in inflammation and the maturation of B cells and is generally known to be produced at sites of acute and chronic inflammation (41). *MMP9* is involved in the breakdown of the extracellular matrix and has roles in tissue metabolic functions, including tumor invasion and metastasis, wound healing, and apoptosis (42). UVB irradiation was reported to increase the expression of *MMP9* in the epidermis, causing apoptosis (43). *CCL2* is a member of the superfamily of secreted proteins involved in immunoregulatory and inflammatory processes. Upregulation of *CCL2* results in skin inflammation and the recruitment of monocytes and macrophages (44, 45). *CXCL8*, a member of the CXC chemokine family, is a major mediator of the inflammatory response and attracts neutrophils, basophils, and T cells in response to an inflammatory stimulus (46). *PTGS2* is regulated by specific stimulatory events, including inflammation or mitogenesis, and acts as a dioxygenase and peroxidase. High expression of *PTGS2* was demonstrated to be associated with cutaneous neoplasms and the shorter survival of patients with skin cancers (47, 48). *LIF* is a pleiotropic cytokine with roles in several different systems, including inflammatory processes and hyperplastic events in the skin, and has been shown to act as a pro- and anti-inflammatory mediator (49, 50).

These seven genes were identified as immune system-related factors and well-studied markers for an inflammatory response in the skin. Additionally, our results revealed that inflammatory and immune response-related cell processes were highly predicted in response to UVB exposure (Figure 2). Several studies reported that UV-induced inflammatory reactions are major risk factors for malignant melanoma development (51, 52). Our study also predicted cancer-related signaling from major cell processes (tumor growth, cancer cell growth, apoptosis, and DNA damage) and diseases (skin cancer, nonmelanoma skin cancer, and metastatic melanoma). This suggests that changes in gene expression caused by UVB exposure might induce an inflammatory reaction leading to skin cancer. Skin diseases, such as psoriasis, dermatitis, and vitiligo, were identified as major diseases associated with solar UVB exposure. These diseases are mainly associated with phototherapy rather than being induced by UVB irradiation (53, 54). Therefore, it can be considered that the expression of the screened genes will also change in response to therapeutic modalities. However, some studies reported that UVB-based phototherapy induced reactive oxygen species, an initiator of the immune response (55, 56).

To evaluate the value of the hub genes, we validated the association between the hub genes and the survival rate of patients with malignant melanoma (Figure 3A). Chronic exposure to UVB is the major cause of melanoma or nonmelanoma skin cancer. Malignant melanoma arises from melanocytes and is the most aggressive and lethal form of skin cancer (29, 57). Furthermore, we exposed NHEKs and NHDFs to UVB for 24 h to validate the biomarkers. The 24 h exposure condition was chosen due to its higher sensitivity in measuring DNA damage and organelle damage assay compared to the immediate exposure condition. Through qRT-PCR experiment, we confirmed a significant increase in the expression patterns of three hub genes (*IL1B*, *IL6*, and *LIF*) in fibroblasts and keratinocytes upon UVB exposure. As a result of predicting the survival rates of patients with cutaneous melanoma based on the gene expression profiles of biomarker candidates, a statistically significant association between the biomarkers and patients with cutaneous melanoma was confirmed, as suggested in other studies (9, 58).

In summary, we explored the adverse effects of UVB exposure on the skin by a meta-analysis and biological network analysis, which identified candidate biomarkers and major signaling mechanisms. As a result of verifying the value of the three hub genes (*IL1B*, *IL6*, and *LIF*) through a survival analysis and qRT-PCR experiment, the possibility of a link between changes in hub genomic profiles resulting from UVB exposure on the skin and malignant melanoma was suggested. However, because only acute exposure data were used for this study, it will be necessary to check whether the proposed biomarkers significantly change their expression even after chronic UVB exposure of the skin. Further studies are needed to consider other types of omics data, such as UVB treatment concentration or period.

## Data availability statement

Publicly available datasets were analyzed in this study. This data can be found at: https://www.ncbi.nlm.nih.gov/geo/, https://www.ncbi.nlm.nih.gov/sra/.

## Ethics statement

Ethical approval was not required for the studies on humans in accordance with the local legislation and institutional requirements because only commercially available established cell lines were used.

## Author contributions

YJ: Formal analysis, Investigation, Software, Visualization, Writing – original draft. H-WN: Data curation, Formal analysis, Investigation, Validation, Writing – original draft. DYS: Investigation, Methodology, Validation, Writing – review & editing. JL: Investigation, Validation, Visualization, Writing – review & editing. JPH: Validation, Visualization, Writing – review & editing. HSK: Conceptualization, Formal analysis, Methodology, Writing – review & editing. SJK: Conceptualization, Formal analysis, Methodology, Writing – review & editing. E-JC: Conceptualization, Data curation, Supervision, Writing – review & editing. CL: Conceptualization, Data curation, Supervision, Writing – review & editing. YDH: Conceptualization, Data curation, Supervision, Writing – review & editing. H-JK: Conceptualization, Supervision, Writing – review & editing. YRS: Conceptualization, Supervision, Writing – review & editing.

## Footnotes

^1^GEO, https://www.ncbi.nlm.nih.gov/geo/, last access date: August 2022.

^2^SRA, https://www.ncbi.nlm.nih.gov/sra/, last access date: August 2022
