# Supplementary material for: Integrative analysis of RNA-sequencing and microarray for the identification of adverse effects of UVB exposure on human skin
Source: Front Public Health. 2024 Feb 20;12:1328089. doi: 10.3389/fpubh.2024.1328089 (PMC10913594; doi:10.3389/fpubh.2024.1328089)
Supplement: Supplementary file 1 [file Data_Sheet_1.docx]

**ㄱSupplementary Table 1. Summary of the datasets used in this study.**

| **Accession** | **Platform** | **Tissue** | **Number of samples**  **(treated/control)** | **Treated concentration** | **Reference** |
| --- | --- | --- | --- | --- | --- |
| GSE45493 | Agilent-014850 Microarray 4 × 44 K | Skin biopsy | 19  (10/9) | 1 MED | (1) |
| GSE41078 | Affymetrix Human Genome U133A 2.0 | Skin biopsy | 20  (10/10) | 2 MED | (2) |
| Kim et al. | Illumina HiSeq 2500 | NHEK | 6  (3/3) | 13 mJ cm−2 | (3) |
| SRP153106 | Illumina HiSeq 2000,  Illumina HiSeq 2500 | NHDF | 12  (6/6) | 0.1 J cm−2 for 1 or 4 h | (4) |
| SRP340060 | Illumina NextSeq 500 | HaCaT | 6  (3/3) | 10 mg cm−2 for 10 times | (5) |
| SRP383038 | Illumina NovaSeq 6000 | HaCaT | 6  (3/3) | 100 mJ cm−2 for 30 min | (6) |
| SRP302398 | HiSeq X Ten | HaCaT | 2  (1/1) | 10 mJ cm−2 for 10 s | (7) |
| GSE54413 | Illumina Genome Analyzer IIx | Skin biopsy | 9  (5/4) | 3 MED | (8) |

MED, minimal erythema dose; NHEK, normal human epidermal keratinocyte; NHDF, normal human dermal fibroblast.

**Supplementary Table 2. Primer sequences for qRT-PCR**

| **Gene** | **REfSEq** | **Forward /Reverse** | **Sequence (5′→3′)** | **Thermocycling condition** |
| --- | --- | --- | --- | --- |
| *IL1B* | NM_000576.3 | Forward | AGG CTG CTC TGG GAT TC | 95°C for 5min, 40 cycles of 95°C for 10 s, 60°C for 15 s, 72°C for 20 s |
|  |  | Reverse | GCC ACA ACA ACT GAC GC |  |
| *CCL2* | NM_002982.4 | Forward | AGT CTC TGC CGC CCT TCT | 48°C for 30 min, 95°C for 10 min followed by 45 cycles of 95°C for 15 s and 60°C for 1 min |
|  |  | Reverse | GTG ACT GGG GCA TTG ATT |  |
| *LIF* | NM_001257135.2 | Forward | ACA GAG CCT TTG CGT GAA AC | 95°C for 5min, 50 cycles of 95°C for 15 s, 60°C for 30 s, and 72°C for 45 s |
|  |  | Reverse | TGG TCC ACA CCA GCA GAT AA |  |
| *GAPDH* | NM_001289745.3 | Forward | ATG GGG AAG GTG AAG GTC G | 95°C for 5min, 40 cycles of 95°C for 10 s, 60°C for 15 s, 72°C for 20 s |
|  |  | Reverse | GGG GTC ATT GAT GGC AAC AA |  |

**Supplementary Table 3. GO enrichment analysis of the upregulated DEGs**

| **Ontology** | **GO term ID** | **Description** | **GeneRatio** | **Adjusted**  ***p*-value** | **Count** |
| --- | --- | --- | --- | --- | --- |
| BP | GO:0032496 | response to lipopolysaccharide | 35/375 | 2.57E-12 | 35 |
| BP | GO:0019221 | cytokine-mediated signaling pathway | 42/375 | 2.57E-12 | 42 |
| BP | GO:0002237 | response to molecule of bacterial origin | 35/375 | 8.46E-12 | 35 |
| BP | GO:0016032 | viral process | 33/375 | 2.02E-08 | 33 |
| BP | GO:0044703 | multi-organism reproductive process | 23/375 | 2.03E-08 | 23 |
| BP | GO:0052548 | regulation of endopeptidase activity | 33/375 | 2.24E-08 | 33 |
| BP | GO:0052547 | regulation of peptidase activity | 34/375 | 2.69E-08 | 34 |
| BP | GO:0044706 | multi-multicellular organism process | 23/375 | 2.79E-08 | 23 |
| BP | GO:0009615 | response to virus | 31/375 | 3.62E-08 | 31 |
| BP | GO:0001819 | positive regulation of cytokine production | 34/375 | 5.63E-08 | 34 |
| CC | GO:0070820 | tertiary granule | 18/378 | 6.76E-07 | 18 |
| CC | GO:0101002 | ficolin-1-rich granule | 19/378 | 6.76E-07 | 19 |
| CC | GO:0034774 | secretory granule lumen | 24/378 | 1.99E-06 | 24 |
| CC | GO:0060205 | cytoplasmic vesicle lumen | 24/378 | 1.99E-06 | 24 |
| CC | GO:0031983 | vesicle lumen | 24/378 | 1.99E-06 | 24 |
| CC | GO:0042581 | specific granule | 15/378 | 3.38E-05 | 15 |
| CC | GO:1904813 | ficolin-1-rich granule lumen | 13/378 | 4.68E-05 | 13 |
| CC | GO:0101031 | chaperone complex | 7/378 | 0.000159 | 7 |
| CC | GO:0070821 | tertiary granule membrane | 9/378 | 0.000483 | 9 |
| CC | GO:0005925 | focal adhesion | 21/378 | 0.002803 | 21 |
| MF | GO:0005125 | cytokine activity | 22/375 | 2.06E-06 | 22 |
| MF | GO:0031072 | heat shock protein binding | 13/375 | 0.000462 | 13 |
| MF | GO:0048018 | receptor ligand activity | 26/375 | 0.001434 | 26 |
| MF | GO:0051087 | chaperone binding | 11/375 | 0.001434 | 11 |
| MF | GO:0030546 | signaling receptor activator activity | 26/375 | 0.001434 | 26 |
| MF | GO:0005126 | cytokine receptor binding | 18/375 | 0.001434 | 18 |
| MF | GO:0045236 | CXCR chemokine receptor binding | 5/375 | 0.00226 | 5 |
| MF | GO:0051082 | unfolded protein binding | 11/375 | 0.00314 | 11 |
| MF | GO:0008009 | chemokine activity | 7/375 | 0.00423 | 7 |
| MF | GO:0004857 | enzyme inhibitor activity | 20/375 | 0.010655 | 20 |

**Supplementary Table 4. GO enrichment analysis of the downregulated DEGs**

| **Ontology** | **GO term ID** | **Description** | **GeneRatio** | **Adjusted**  ***p*-value** | **Count** |
| --- | --- | --- | --- | --- | --- |
| BP | GO:0006805 | xenobiotic metabolic process | 11/179 | 1.81E-05 | 11 |
| BP | GO:0071466 | cellular response to xenobiotic stimulus | 12/179 | 0.000102 | 12 |
| BP | GO:0009410 | response to xenobiotic stimulus | 17/179 | 0.000391 | 17 |
| BP | GO:0042537 | benzene-containing compound metabolic process | 5/179 | 0.003436 | 5 |
| BP | GO:0007369 | gastrulation | 10/179 | 0.007877 | 10 |
| BP | GO:0007163 | establishment or maintenance of cell polarity | 10/179 | 0.022149 | 10 |
| BP | GO:0030177 | positive regulation of Wnt signaling pathway | 8/179 | 0.023445 | 8 |
| BP | GO:0071230 | cellular response to amino acid stimulus | 6/179 | 0.023445 | 6 |
| BP | GO:0090263 | positive regulation of canonical Wnt signaling pathway | 7/179 | 0.023445 | 7 |
| BP | GO:0042178 | xenobiotic catabolic process | 4/179 | 0.023445 | 4 |
| CC | GO:0098644 | complex of collagen trimers | 4/186 | 0.014438 | 4 |
| MF | GO:0016712 | oxidoreductase activity, acting on paired donors, with incorporation or reduction of molecular oxygen, reduced flavin or flavoprotein as one donor, and incorporation of one atom of oxygen | 5/180 | 0.017825 | 5 |
| MF | GO:0004364 | glutathione transferase activity | 4/180 | 0.023618 | 4 |

**Supplementary Table 5. Centrality information of top 20 genes.**

| **Gene symbol** | **Betweenness centrality** | **Degree** |
| --- | --- | --- |
| *IL1B* | 0.2706915 | 57 |
| *IL6* | 0.2301223 | 56 |
| *MMP9* | 0.0797234 | 28 |
| *CCL2* | 0.0740171 | 32 |
| *CXCL8* | 0.0723333 | 31 |
| *PTGS2* | 0.0705397 | 32 |
| *LIF* | 0.0315344 | 14 |
| *MMP1* | 0.0193871 | 16 |
| *NR4A3* | 0.0088221 | 5 |
| *MMP3* | 0.0075839 | 21 |
| *CDKN1A* | 0.0044633 | 9 |
| *AREG* | 0.0033267 | 8 |
| *CXCL2* | 0.0026879 | 13 |
| *TIMP1* | 0.0024693 | 10 |
| *SOD2* | 0.0019659 | 6 |
| *S100A9* | 0.0019494 | 14 |
| *NR4A2* | 0.0016455 | 8 |
| *CXCL1* | 0.0013723 | 14 |
| *TNFAIP6* | 0.000965 | 9 |
| *S100A8* | 0.000961 | 15 |


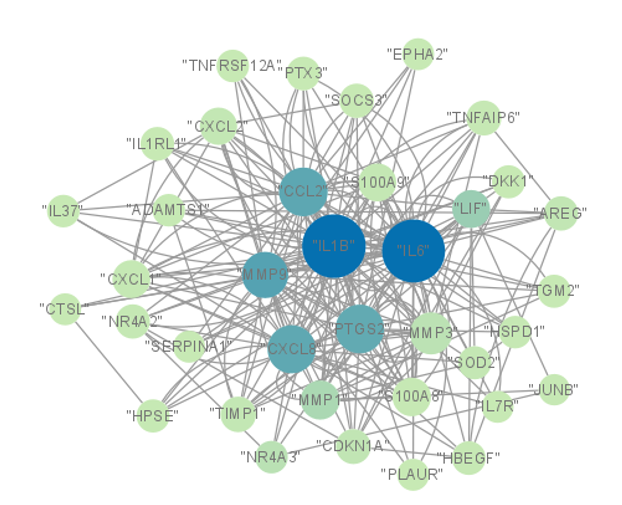


**Supplement Figure 1. a gene−gene interaction network by Cytoscape.** The node color was from pale green to blue, representing betweenness centrality and the corresponding degree gradually larger.


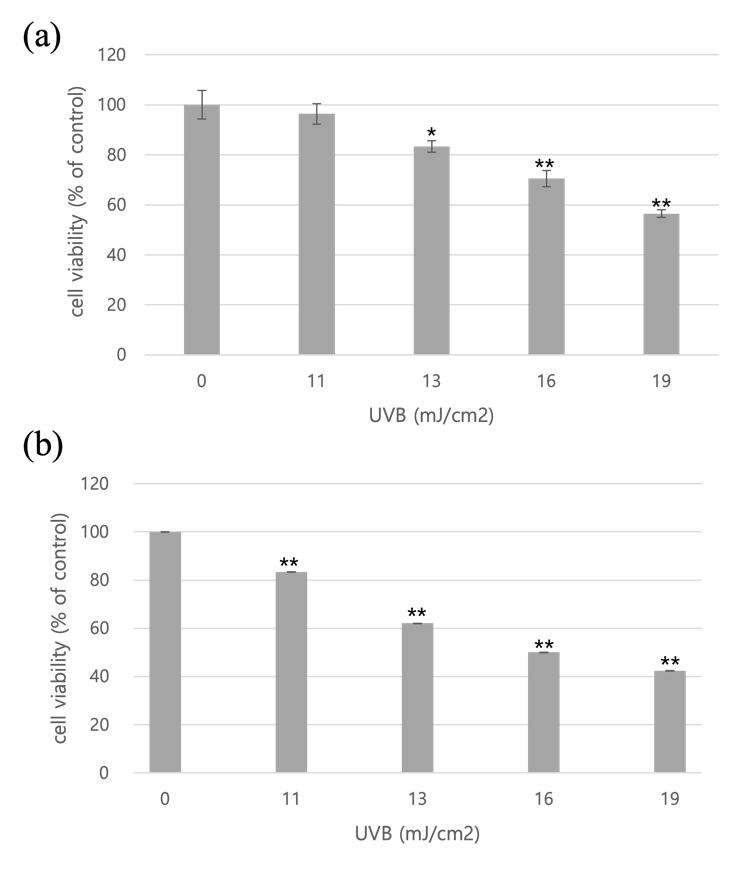


**Supplement Figure 2. Cytotoxicity of UVB on Skin. a)** CCK-8 assay result in the NHEKs exposed to UVB irradiation. **b)** the 3-(4,5-dimethylthiazol-2-yl)-2,5 diphenyltetrazolium bromide assay result in the NHDFs exposed to UVB irradiation. Error bars represent ± SEM. * and ** indicate *p*-value <0.05 and <0.01, respectively.

**References**

1. Bustamante M, Hernandez-Ferrer C, Tewari A, Sarria Y, Harrison GI, Puigdecanet E, et al. Dose and time effects of solar-simulated ultraviolet radiation on the in vivo human skin transcriptome. Br J Dermatol. 2020;182(6):1458-68.

2. Kennedy Crispin M, Fuentes-Duculan J, Gulati N, Johnson-Huang LM, Lentini T, Sullivan-Whalen M, et al. Gene profiling of narrowband UVB-induced skin injury defines cellular and molecular innate immune responses. J Invest Dermatol. 2013;133(3):692-701.

3. Kim SJ, Na H-W, Jang Y, Shin DY, Choi H, Kim H-J, et al. Network analysis to understand side effects of UVB on skin through transcriptomic approach. Molecular & Cellular Toxicology. 2022;18(4):457-67.

4. Kim HS, Kim YJ, Kim SJ, Kang DS, Lee TR, Shin DW, et al. Transcriptomic analysis of human dermal fibroblast cells reveals potential mechanisms underlying the protective effects of visible red light against damage from ultraviolet B light. J Dermatol Sci. 2019;94(2):276-83.

5. Li S, Dina Kuo HC, Wang L, Wu R, Sargsyan D, Kong AN. UVB Drives Metabolic Rewiring and Epigenetic Reprograming and Protection by Sulforaphane in Human Skin Keratinocytes. Chem Res Toxicol. 2022;35(7):1220-33.

6. Jiang H, Zhou X, Chen L. Asiaticoside delays senescence and attenuate generation of ROS in UV‑exposure cells through regulates TGF‑beta1/Smad pathway. Exp Ther Med. 2022;24(5):667.

7. Kang W, Son B, Park S, Choi D, Park T. UV-Irradiation- and Inflammation-Induced Skin Barrier Dysfunction Is Associated with the Expression of Olfactory Receptor Genes in Human Keratinocytes. Int J Mol Sci. 2021;22(6).

8. Dawes JM, Antunes-Martins A, Perkins JR, Paterson KJ, Sisignano M, Schmid R, et al. Genome-wide transcriptional profiling of skin and dorsal root ganglia after ultraviolet-B-induced inflammation. PLoS One. 2014;9(4):e93338.
